# Environmental Tobacco Smoke and Cardiovascular Disease

**DOI:** 10.3390/ijerph16010096

**Published:** 2018-12-31

**Authors:** Sydne I. DiGiacomo, Mohammad-Ali Jazayeri, Rajat S. Barua, John A. Ambrose

**Affiliations:** 1 Department of Internal Medicine, University of Kansas Medical Center, Kansas City, KS 66160, USA; sDiGiacomo@kumc.edu; 2 Department of Cardiovascular Medicine of Cardiovascular Diseases, University of Kansas Medical Center, Kansas City, KS 66160, USA; mjazayeri@kumc.edu; 3 Division of Cardiovascular Medicine, Kansas City VA Medical Center, Kansas City, MO 64128, USA; 4 Division of Cardiovascular Medicine, University of California San Francisco, Fresno, CA 93701, USA; jamambrose@yahoo.com

**Keywords:** environmental tobacco smoke, cardiovascular disease, atherosclerosis, thrombosis, plaque biology, cigarettes, smoking cessation, public health

## Abstract

Environmental tobacco smoke (ETS) and its sequelae are among the largest economic and healthcare burdens in the United States and worldwide. The relationship between active smoking and atherosclerosis is well-described in the literature. However, the specific mechanisms by which ETS influences atherosclerosis are incompletely understood. In this paper, we highlight the definition and chemical constituents of ETS, review the existing literature outlining the effects of ETS on atherogenesis and thrombosis in both animal and human models, and briefly outline the public health implications of ETS based on these data.

## 1. Introduction

Environmental tobacco smoke (ETS) remains a major public health concern in the United States. While the percentage of Americans who actively smoke has decreased in recent years, the economic and healthcare effects of ETS remain substantial, both in the United States (US), and throughout the rest of the world. A 2014 report from the US Surgeon General estimated lost productivity caused by ETS exposure, specifically secondhand smoking (SHS), accounted for $5.6 billion US dollars in 2006 alone [1]. In this review, we aim to highlight the definition and chemical constituents of ETS, review the existing literature outlining the effects of ETS on atherogenesis and thrombosis in both animal and human models, and briefly discuss the public health implications of ETS.

## 2. Methods

This literature review was conducted using PubMed as the primary search engine to obtain relevant studies on the effects of ETS on atherogenesis and thrombosis, in both animal and human models. Search terms included: environmental tobacco, second hand smoke exposure, second hand tobacco exposure, tobacco and atherogenesis, tobacco and thrombosis, and environmental tobacco biochemical properties. A complete list of search terms is provided in the Supplementary Index. Inclusion criteria were limited to studies published in English and studies that were relevant to the topic of the paper. Selected studies included both clinical and basic science research publications, review papers, as well as federally funded reports published by US national health agencies. Case reports were excluded from consideration for this review. A total of 57 studies were included, among which 40 were experimental studies comprised of 28 human studies, 11 animal studies, and one biochemical study. The remaining studies consisted of seminal reviews, professional societal consensus statements, systematic reviews, and federally funded reports.

## 3. ETS: Definition and Physical/Biochemical Properties

ETS originates from the smoldering end of cigarettes in between puffs, also referred to as sidestream smoke (SS), and from smokers’ exhaled smoke. The smoke inhaled by the smoker is referred to, in contrast, as mainstream smoke (MS). ETS is comprised of 15% MS and 85% SS. Toxic compounds such as ammonia, volatile amines and nitrosamines, nicotine decomposition products, and aromatic amines are found in higher concentrations in undiluted SS when compared to undiluted MS. ETS contains aerosolized chemical constituents that exist in either hydrophilic or hydrophobic vapor phases. The hydrophilic vapor phase constituents of ETS are more easily absorbed in the upper respiratory tract, while hydrophobic constituents are more likely to enter the lung. Particles smaller than 2.5 μm are referred to as respirable suspended particles (RSPs) by the US National Research Council Committee on Passive Smoking and can be inhaled deep into the lung parenchyma. Depending on the degree of air dilution of SS, the concentration of particles in ETS can range from a few micrograms to 300–500 mg per cubic meter. The biological markers most useful for assessing recent exposure to ETS are nicotine and its metabolite, cotinine. Additionally, measurement of RSPs in the environment has proven to be a useful surrogate for ETS exposure [2]. Table 1 summarizes clinically relevant components of ETS and corresponding biomarkers of exposure.

## 4. Physiology of Atherogenesis and Thrombosis

Both atherogenesis and thrombosis are sentinel events preceding arterial occlusion, the mechanism by which myocardial infarctions and strokes occur. Atherogenesis is the process by which atherosclerosis (build-up of arterial wall plaques and subsequent arterial hardening) begins to develop within arterial walls. Affected arterial systems include coronary arteries, cerebral arteries, renal arteries, peripheral arteries and the aorta. Initiation of atherosclerosis begins with molecular changes that occur within the innermost layer of the arterial wall, known as the intima. The intima is lined with a single layer of cells called endothelial cells, which separate the lumen of the artery from the sub-intimal layers known as the media and adventitia. Endothelial injury (caused by conditions such as hypertension, diabetes, hyperlipidemia, and toxins such as those from tobacco smoke) and subsequent dysfunction promote deposition of lipoproteins into the vessel wall. Oxidation of lipoproteins is followed by adhesion of monocytes to the lesion. Migration of monocytes into the sub-intimal territory and subsequent “consumption” of lipoproteins gives rise to foam cells, which collectively contribute to the “fatty streak” within the intimal layer of the arterial wall. Inflammatory mediators and smooth muscle cells migrate from into the intima to promote formation of an atheromatous plaque [3]

Thrombosis is the process by which a thrombus [blood clot] is formed and causes occlusion of the vessel in which it is formed. Disruptions in hemostasis occur via several mechanisms, including endothelial cell injury, stasis or turbulence of blood flow, and hypercoagulability of blood. Primary hemostasis refers to the process by which platelets adhere to sites of endothelial injury and aggregate to form a platelet plug. This process is followed by secondary hemostasis, which involves progression through a coagulation cascade to generate cross linked fibrin products. Disruption of atheromatous plaques formed within arterial walls causes exposure of thrombogenic material and rapid formation of thrombus, which in turn increases the size of the intimal plaque and causes hemodynamically significant occlusion of the effective luminal area [3].

## 5. Epidemiology

A 10-year prospective study of nonsmoking, community dwelling women in the US (*n* = 695) aged 50–79 years whose husbands were never, former, or current smokers found a higher total and age-adjusted death rate due to ischemic heart disease in wives of current or former smokers, as compared to those whose husbands never smoked. After adjustment for cardiovascular disease (CVD) risk factors, the relative risk for those women married to current and former smokers was 14.9, as compared to wives of never smokers [4]. Similarly, the results of a 15-year prospective study of nonsmoking Japanese women (*n* = 91,540) classified during follow-up by the smoking status of their husbands have been reported. Among women married to men smoking ≥20 cigarettes daily (*n* = 25,461), the standardized mortality rate ratio from ischemic heart disease was estimated to be 1.31 (90% CI 1.06–1.63, *p* = 0.019) compared with nonsmoking women whose husbands did not smoke [5]. In the Multiple Risk Factor Intervention Trial (MRFIT), there was a roughly two-fold increase in the risk of CHD morbidity and mortality among non-smokers exposed to ETS [6]. Based on these studies, as well as several meta-analyses, it was suggested that although the dose of cigarette smoke delivered to passive smokers is 1% of that delivered to active smokers, the relative risk (RR) of coronary artery disease for passive smokers (RR 1.31 compared to non-exposed, 95% CI 1.21–1.41) approaches 40% of the risk of active smokers (RR 1.78 compared to nonsmokers, 95% CI 1.31–2.44) [7]. In the Cardiovascular Risk in the Young Finns Study (1980), the relative risk of developing carotid plaque in those patients for whom one or both parents were smokers was 1.7 (95% CI 1.0–2.8, *p* = 0.04) [8]. The results of this prospective study, when adjusted for childhood and adulthood lipid profiles and blood pressure measurements, suggest that ETS during childhood may be independent of other risk factors for CVD, via mechanisms other than those related to lipid and blood pressure profiles, inflammation, and body mass index (BMI) [8].

## 6. ETS and Atherosclerosis: Clinical and Experimental Observations

**Figure 1:**
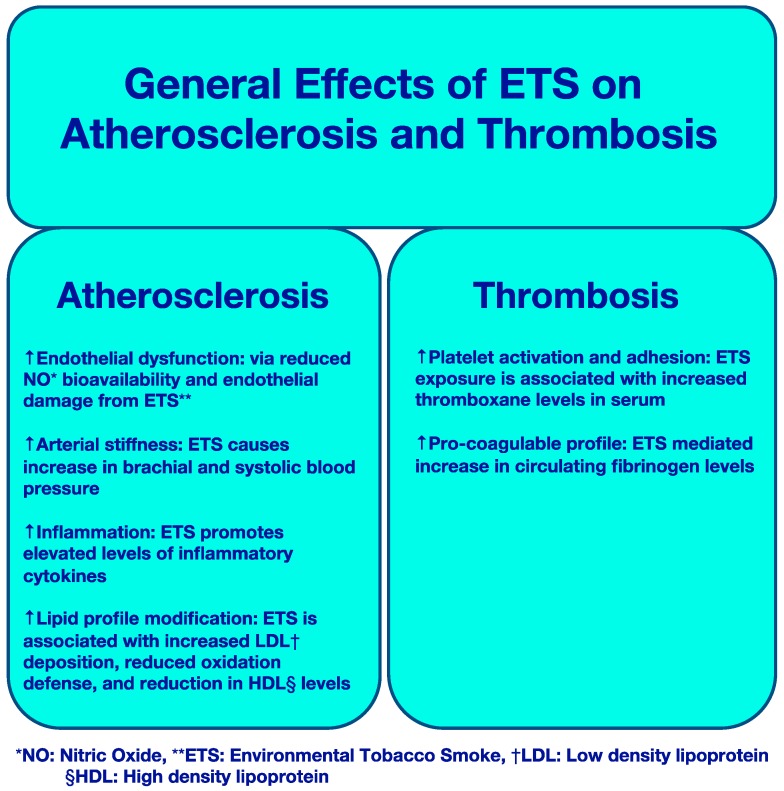
Effects of ETS on Atherosclerosis and Thrombosis.

ETS has been shown to cause pro-atherogenic changes of multiple cardiovascular parameters in both the clinical and experimental settings. Figure 1 summarizes ETS effects on both atherosclerosis and thrombosis.

### 6.1. Effects on Endothelial Function

Impairment of endothelial vasodilatory function is one of the earliest manifestations of atherosclerotic changes in a blood vessel. Nitric oxide (NO) production or bioavailability from the endothelial cells mediates endothelial dependent vasodilation (EDV) [9]. A study exposing 10 healthy non-smokers to 30 min of ETS demonstrated decreased flow mediated dilation (FMD) for 1 h post-exposure, followed by FMD recovery at 2.5 h. However, there was persistent elevation of both endothelial microparticles and vascular endothelial growth factor levels at 24 h, indicating functional endothelial impairment [10]. Similarly, coronary flow velocity reserve (ratio of hyperemic to basal coronary flow velocity, as determined by Doppler ultrasound tracings), a surrogate marker of EDV, was found to be significantly reduced in non-smoking Japanese males after exposure to ETS (4.4 ± 0.91 before passive smoking vs. 3.4 ± 0.73 after passive smoking, *p* < 0.001) [11]. Furthermore, it has been reported that the extent of EDV impairment is similar between ETS and active smokers [11,12]. Another prospective study compared endothelial cells and flow-mediated dilation via direct endothelial cell sampling in healthy, young individuals divided into 3 groups: active smokers, passive smokers, and non-smoker controls [13]. The authors found ETS exposure was associated with reduced endothelial NO synthase (eNOS) (37% reduced expression, *p* = 0.04) and activated, phosphorylated eNOS levels (65% reduced expression, *p* = 0.02), as compared to non-smoking, age-matched controls [13].

Similar findings have been observed in animal models as well. EDV in aortic rings of hypercholesterolemic rabbits exposed to ETS was impaired in one study, a change reversible by administration of L-arginine, the precursor to NO [14]. In rats consuming a high cholesterol diet, ETS exposure was shown to produce significantly reduced endothelium-dependent relaxation when compared to controls receiving high cholesterol diet alone. Both high cholesterol diet and ETS exposure led to atherogenesis as well [15].

In addition to functional changes, ETS may directly impair the viability of endothelial cells and has been shown to cause direct endothelial cell injury. In one study, exposure to 20 min of SHS was associated with increased circulating levels of endothelial cell carcasses [16]. Rat endothelial cells exposed to 6 weeks of ETS (6 h per day, 5 days per week) were found to contain abnormal cytoplasmic vacuoles and compromised microtubule bundles, as well as disrupted junctional complexes between adjacent cells [17]. Such injuries in endothelial cells leads to increased vascular permeability and promotes atherosclerosis [7]. A more recent study by Heiss et al. demonstrated that brief exposure to real-world levels of ETS led to sustained vascular injury, characterized by mobilization and proliferation of dysfunctional endothelial progenitor cells (EPCs) [10].

### 6.2. Effects on Arterial Stiffness

In addition to causing impaired EDV in arteries, ETS also increases arterial stiffness. In one study, healthy male subjects exposed to ETS from 15 cigarettes in an unventilated room for 1 h demonstrated a significant increase in the augmentation index, a marker of arterial stiffness (*p* < 0.001). About half of the increase had occurred at 15 min, and it reached steady state after 30 min, a change which was associated with increases in both brachial and aortic systolic blood pressure [18]. In a cross-sectional epidemiologic study of adults, those with a body mass index (BMI) of ≥ 27.1 kg/m^2^ and intimal medical thickness (IMT) ≥ 0.707 mm who were chronically exposed to ETS exposure at home, work, and other places had a significant increase in carotid stiffness index as a function of daily hours of ETS exposure (linear trend = 0.04 for both BMI and IMT, *p* = 0.001 for BMI interaction, *p* = 0.003 for IMT interaction) [19].

### 6.3. Inflammation

An ongoing inflammatory response is an essential component in the initiation and progression of atherosclerosis. In both adults and children, ETS exposure has been associated with elevated inflammatory markers. Acute phase proteins such as complement 3c, haptoglobin, and alpha 1-acid glycoprotein are significantly increased in boys exposed to 11 or more cigarettes per day [20]. Adult nonsmokers exposed to ETS have increases in leukocyte count (*p* < 0.005), release of reactive oxidants by stimulated neutrophils (*p* < 0.005), and chemotaxis (*p* < 0.001) after 3 h of exposure [21]. Men and women exposed to ETS > 3 days/week demonstrated higher levels of C-reactive protein (CRP) (*p* = 0.03), homocysteine (*p* = 0.002), oxidized low-density lipoprotein (LDL) cholesterol (*p* = 0.03), and white blood cell counts (*p* = 0.02), after adjustment for potential confounders, when compared to those not exposed to ETS [22]. In work by Adams et al., venous endothelial cells obtained from human subjects with ETS exposure (i.e. passive smokers) showed increased activity of inflammatory marker nuclear factor-kB (NF-kB), when compared to non-exposed controls (*p* = 0.007), while NF-kB levels were found to be similar among passive and active smokers (*p* = 0.47) [13]. A Multi-Ethnic Study of Atherosclerosis (MESA) sub-study evaluated over 5000 nonsmoking adults exposed to ETS from the prospective cohort study of ethnically diverse subjects. Exposure was defined by self report and urine cotinine levels. It was found that ≥ 12 h of ETS exposure per week was associated with higher levels of the inflammatory marker high sensitivity C-reactive protein (hsCRP) (geometric mean ratio, 1.13; 95% CI 1.02–1.26) after full adjustment for potential confounders [23].

In mouse models, pro-inflammatory cytokine production has been shown to increase with ETS exposure. Zhang et al. demonstrated that 120 min of SS exposure per day was sufficient to increase circulating levels of interleukin-1b (IL-1B) (*p* < 0.05), interleukin-6 (IL-6) (*p* < 0.05), and tumor necrosis factor-alpha (TNF-α) (*p* < 0.05), when compared to both non-exposed sham controls and 60-min of SS exposure [24]. The immune response has also been implicated in atherosclerosis in studies of ETS exposure in mice. Yuan et al. showed in their study increased levels of interleukin-12 (IL-12), and subsequently TNF-α expression, following ETS exposure. They further demonstrated that ETS stimulated monocyte chemoattractant protein-1 (MMP-1) secretion, which is involved in chemotaxis and migration of monocytes into the arterial wall to form precursors to atherosclerotic plaque [25].

### 6.4. Modification of Lipid Profile

ETS may promote atherosclerosis, in part, by its effects on lipid profile. In one human study by Valkonen et al., a short period (30 min) of ETS exposure from 16 cigarettes was shown to accelerate lipid peroxidation and LDL modification. In the same study, ETS exposure was associated with an increased accumulation of LDL cholesterol in human macrophages. The authors demonstrated that ETS decreased serum antioxidant defense (33% decrease in serum ascorbic acid, *p* < 0.001), with a simultaneous 19% decrease in LDL resistance to copper-mediated oxidation (*p* < 0.01) [26].

A study evaluated aortic lesion development and cholesterol deposition in apolipoprotein E (apoE) deficient mice exposed to cigarette SS. Filtered ambient air exposed mice were used as a control group, and experimental groups were assigned to SS exposure in a whole-body chamber for 6 h per day, 5 days per week, for a total of 7, 10, and 14 weeks, respectively. Researchers found increases in grossly discernible lesions covering the intimal area of the aorta in SS exposed mice at all intervals, along with increased levels of cholesterol in aortic tissues of SS exposed mice (at 14 weeks: 33 ± 11% of the intima covered by lesions in ETS exposed group vs. 10 ± 8% in control group, *p* < 0.001) [27].

Furthermore, ETS exposure has been shown to be associated with decreased athero-protective HDL levels. In adults, a 1-mg/dL decrease in HDL level is associated with a 2% to 3% increase in coronary heart disease risk. Female subjects exposed to ETS for at least 6 h per day, 4 days per week, for at least 6 consecutive months showed a significant decrease in HDL-C, HDL_2_, and apolipoprotein A-I levels compared to nonsmokers (*p* < 0.05) [28]. Values were not significantly different between ETS-exposed subjects and active smokers. In another study, the same investigators exposed 12 male subjects to 6 h of ETS at concentrations similar to those found in a bar and found that HDL levels at 8, 16, and 24 h later were significantly reduced from baseline at all 3 follow-up time points (18%, 14%, and 13% reductions, respectively) [7,29]. Similarly, mean serum HDL levels were found to be significantly lower among children exposed to ETS in the household, as compared to children who were not exposed to ETS (38.7 ± 1.2 vs. 43.6 ± 1.2 mg/dL, *p* = 0.005), an association which remained significant following multivariate regression analysis. LDL levels and triglyceride levels were not significant different between the two groups [30].

### 6.5. ETS and Progression of Atherosclerosis

ETS exposure has been found to be associated with accelerated progression of atherosclerosis. A population-based cohort study of middle-aged adults found that ETS with a mean exposure time of 10 h per week for 3 years was associated with a 20% increase in progression of atherosclerosis (as measured by an interval change in common carotid intimal medial thickness) when compared to non-smokers without ETS exposure [31]. In a retrospective study of patients undergoing coronary arteriography, ETS exposure in Chinese women who had never smoked cigarettes was significantly associated with severity of coronary artery disease after adjustment for other major risk factors [32]. These data suggest that the number of stenotic arteries increase with the amount of exposure to ETS from the husband [32]. Similar findings have been observed in animal models as well. ApoE−/− mice exposed to ETS from 2 cigarettes for 15 min per day over 21 or 42 days were found to have atherosclerotic lesions that increased in size by 76% and 156%, respectively, compared with unexposed mice [33]. In a rabbit model of human atherosclerosis, ETS exposure over 10 weeks was associated with significant increases in atherosclerotic burden in the aorta and pulmonary artery [34].

ETS may also contribute to atherosclerotic disease progression via its effects on matrix metalloproteinases (MMPs). MMPs are responsible for weakening the plaque fibrous cap and contribute to destabilization and rupture of atherosclerotic plaques, leading to thrombus formation. Indeed, saphenous veins isolated from women with ETS exposure showed significantly higher MMP-2 and MMP-9 gene expression (*p* < 0.05), and subsequent studies have shown increased MMP gene expression to be associated with decreased saphenous vein graft patency [35]. Nicotine, at concentrations typically found in ETS exposure, upregulates collagenase I, a type of matrix metalloproteinase found in human artery smooth muscle cells [7].

## 7. ETS and Thrombosis: Clinical and Experimental Observations

ETS may also contribute to atherosclerotic progression through its effects on promoting thrombosis.

### 7.1. ETS and Platelet Function

Platelet activation and thrombosis at sites of atheromatous plaque disruption play a key role in the pathophysiology of acute coronary events. In one study, 20 min of ETS exposure was associated with increased platelet activation in healthy male nonsmokers to an extent comparable to that of actively smoking 1–2 cigarettes [36]. Thromboxane, another marker of platelet activation, is also increased in subjects exposed to ETS, in some cases to levels observed in active smokers [37]. The preponderance of data suggests a non-linear relationship between ETS and thrombogenesis [38]. Notably, a study which exposed 10 healthy nonsmoking males to 20 min of ETS revealed that ETS affected endothelial cell counts (mean values: 2.8 ± 0.9 before exposure and 3.7 + 1.1 after exposure, mean difference 0.9, 95% CI: 0 −1.8) and platelet aggregate ratios (mean values: 0.87 ± 0.06 before exposure and 0.78 ± 0.07 after exposure to passive smoking, mean difference 0.09, 95% CI: 0.03—0.15) similarly to those of active smokers [36].

### 7.2. ETS and Coagulation Cascade

Fibrinogen is a known acute-phase reactant and risk factor for thrombosis, which has been found to be elevated in relation to ETS exposure. Japanese people exposed to ETS had a higher mean covariate-adjusted fibrinogen level than nonsmokers not exposed to ETS. The degree of fibrinogen increase in ETS-exposed individuals accounted for 62% of the difference between active smokers and non ETS-exposed nonsmokers [39]. Similarly, teenagers living with a smoker at home have higher fibrinogen levels than those living in a smoke-free home [40]. Beyond fibrinogen, elevated circulating levels of coagulation factors such as thrombin and tissue factor have been found in the plasma of smokers, as compared to nonsmokers [41].

### 7.3. Effect on Heart Rate and Heart Rate Variability

Reduction in heart rate variability has been associated with an increased risk of ventricular tachyarrhythmias in patients following myocardial infarction (MI) and in those with chronic heart failure (HF) [42]. In a study performed in an airport smoking lounge, it was reported that two hours of exposure was associated with a 12% reduction in heart rate variability [43]. Two hours later when the subjects were out of the smoking room, the heart rate variability returned to baseline. The study suggested that in a vulnerable individual, ETS may increase the risk of fatal tachyarrhythmias. It has been proposed that this effect of ETS on heart rate variability may be mediated by the fine particles in ETS [7,43].

Table 2 summarizes studies regarding the acute effects of ETS on clinical parameters in healthy subjects.

### 7.4. ETS and Anginal Symptoms

The effects of ETS on patients with chronic stable angina have been previously reported. One study found subjects exposed to low levels of carbon monoxide experienced anginal pain when carboxyhemoglobin levels in serum were measured at 2–4% [3]. This finding suggests that the low threshold for onset of angina in patients exposed to ETS may be related to a decrease in oxygen carrying capacity due to an increased affinity of CO for hemoglobin, presumed vasoconstriction and increase in rate-pressure product [44].

### 7.5. ETS and Infarct Size

ETS exposure has been shown to impact myocardial infarct size in the experimental setting. A study involving rats exposed to 6 weeks of ETS for 6 h per day, 5 days per week at levels observed in bars, demonstrated infarcts twice as large in the ETS group as compared to the unexposed group after an infarct was induced [44]. Similarly, exposure to ETS in the neonatal to adolescent period for 12 weeks significantly increased experimentally induced infarct size, especially in female rats [44].

## 8. Factors and Mechanisms Responsible for ETS-Mediated Cardiovascular Dysfunction

Over 4000 chemical compounds exist in cigarette smoke. Of these compounds, polycyclic aromatic hydrocarbons have been shown to accelerate atherosclerosis, via production of inflammatory interleukins, and contribute to foam cell formation. Carbon monoxide has not been found to cause atherosclerosis or thrombus formation.

Despite being the addictive component of tobacco smoke, there are conflicting data on the role of nicotine on NO availability and EDV. There appears to be an insignificant effect of nicotine on thrombo-hemostatic factors of the clotting cascade [9]. Similarly, nicotine exposure does not appear to be a primary cause for ETS exposure related to increases in atherosclerotic lesions, as similar effects have been observed in ETS exposure with nicotine-free cigarettes. This strongly suggests that components other than nicotine in ETS are responsible for promoting atherosclerosis [50].

The quintessential role of free-radical mediated oxidative stress in atherosclerosis has been elucidated in both active and now passive smoking research. The volatile compounds in side stream smoke, notably the polycyclic aromatic hydrocarbons, are known to initiate endothelial dysfunction through down regulation of NO bioavailability and synthesis. Free radicals are thought to form from (1) the gas or tar phase of cigarette smoke; (2) circulating or in situ-activated macrophages and neutrophils; and (3) endogenous sources of reactive oxygen species such as uncoupled eNOS, xanthine oxidase, and the mitochondrial electron transport chain [9].

## 9. Public Health Implications of ETS-Mediated Cardiovascular Disease

Environmental tobacco smoke adversely impacts both children and adults, and serves as a major economic burden to the US healthcare system. Children exposed to environmental tobacco often develop pulmonary and upper respiratory tract infections, and they are at increased risk of reactive airway diseases such as asthma and bronchitis. Studies have also linked ETS exposure in the home environment to the development of middle ear infections and sudden infant death syndrome [51]. From a cardiovascular perspective, it is estimated that ETS accounts for as many as 62,000 annual ischemic deaths in the US alone [16]. The establishment of morbidity and mortality conferred by ETS exposure in the literature has catalyzed public health policies to change in favor of reducing exposure. In 1988, the first ban was placed on smoking with a restriction to two hours or less of use. Over time, both state and federal legislation limiting ETS have led to changes in smoking behavior. One systematic review across Australia, Germany, United States and Canada found that totally smoke-free workplaces were associated with overall reductions in the prevalence of smoking: 3.8% (95% confidence interval 2.8% to 4.7%) and 3.1 (2.4 to 3.8) fewer cigarettes smoked per day per continuing smoker. [52]. Widespread policy changes as well as a cultural shift in tobacco perception are necessary for meaningful reductions in ETS-related morbidity and mortality.

## 10. Emerging Areas in the Study of ETS

Thirdhand smoke (THS) is residual tobacco smoke that clings to indoor surfaces, and re-emission of gases and resuspension of particles from contaminated surface materials after active smoking has ceased [53]. These are gases and particles, which become embedded in materials from walls to furniture to toys & other household items. Importantly, these residual substances can react, re-emit, and/or resuspend in an environment long after active smoking has ended [53]. Recently published proceedings from previous experiments performed at Philip Morris Inc. demonstrate persistence of high concentrations of nicotine and 4-(methylnitrosamino)-1-(3-pyridyl)-1-butanone (NNK), over 100 days after cigarette smoke exposure was discontinued in a controlled environment [54]. Notably, the amount of NNK recovered at the end of the study was 170% of what was initially generated. In another study it was shown that nicotine from THS could react with a commonly found indoor pollutant to produce tobacco-specific nitrosamines [55]. The potential biological and health effects of THS are just beginning to be uncovered and reported in the scientific literature. In vitro assay and in vivo animal model investigations demonstrate that THS exposure causes DNA damage, alters wound healing, upregulates inflammatory cytokines, and down-regulates anti-inflammatory cytokine activity [56]. THS-exposed mice exhibit an enhanced platelet aggregation and secretion response, as well as enhanced integrin GPIIb-IIIa activation [57]. In mice, THS exposure also increases circulating levels of triglycerides and LDL, while decreasing HDL levels significantly [56]. Over time, all of these effects in combination can adversely impact cardiovascular health. Additional studies are needed to elucidate the full extent of THS effects on the cardiovascular system.

E-cigarettes, also known as “e-cigs” or electronic nicotine delivery systems, have been gaining popularity over the past decade. E-cigarettes differ from traditional cigarettes and other combustible tobacco products in that they do not produce smoke by burning tobacco. Instead, they heat a solution (e-liquid) that usually contains nicotine, propylene glycol or vegetable glycerin, and flavorings to generate an aerosol that the user inhales [58]. E-cigarettes produce an aerosol with fewer chemical compounds than that found in conventional cigarettes [58]. Exhaled e-cigarette vapors have been shown to contain nicotine, glycerine, propylene glycol, formaldehyde, acetaldehyde, polycyclic aromatic hydrocarbons, metals, and ultra-fine particles [59]. Depending on the type of e-cigarette device used, the concentration of nickel, silver, and formaldehyde can be higher in e-cigarette vapors than the secondhand smoke of conventional cigarettes [59]. Scientific evidence on the health impact of nonuser exposure to chemicals from e-cigarettes is limited. However, long-term exposure to nicotine, formaldehyde, heavy metals, and ultra-fine particles is associated with adverse cardiovascular outcomes [60]. Although more research is required, current evidence regarding passive exposure to e-cigarette vapors shows the potential for harmful cardiovascular effects.

## 11. Conclusions

All of the above data suggest ETS exposure promotes athero-thrombosis via endothelial dysfunction, inflammation, platelet adhesion, and plaque instability. The toxic constituents of side stream smoke (the primary component of ETS) are responsible for significant cardiovascular morbidity and mortality through generation of a pro-atherogenic milieu. The mechanisms involved are complex and target mainly the endothelium and several different molecules/blood cells involved in coagulation, leading to a relative hypercoagulable state. As a consequence, ETS is estimated to be responsible for at least 33,000 deaths annually, based on the 2014 US Surgeon General’s Report [1]. At a societal level, the ultimate goal must be zero ETS exposure, with progress being made on this front through legislative efforts resulting in 100% smoke-free policies in workplaces and public spaces. Informed individuals and groups can further promote such efforts in their own locales, with the ultimate goal of eliminating exposure to tobacco in all its various forms.

## Figures and Tables

**Table 1:** Clinically Relevant Components of ETS and Corresponding Biomarkers of Exposure.

Constituent—Chemical Group	Concentration	Human Biomarkers
Acetaldehyde—Volatile Organic Compound	200–300 µg/m^3^	Tissue, Blood N2-ethylidene-dGuo levels
Formaldehyde—Volatile Organic Compound	100–140 µg/m^3^	Tissue, Blood N6-hydroxymethyl deoxyadenosine adducts
Nicotine—Tobacco Alkaloid	10–100 µg/m^3^	Total nicotine equivalents (TNE) * Blood, Salivary or Urinary Cotinine level
1,3-Butadiene—Volatile Organic Compound	20–40 µg/m^3^	Urine Monohydroxybutenyl mercapturic acid (MHBMA)
Benzene—Polyaromatic Hydrocarbon	15–30 µg/m^3^	Urine C,S BAP-tetrol Urine 3-hydroxy-BAP Urine 1-Hydroxypyrene Urine PheT
NNK (nicotine-derived nitrosamine ketone)—Tobacco-specific nitrosamine	0.2–29.3 µg/m^3^	Urine NNK Urine NNAL Urine NNAL-N-Gluc Urine NNAL-O-Gluc
Carbon Monoxide—Variable Gas	5–20 µg/m^3^	Exhaled CO ([CO] in ppm) Blood carboxyhemoglobin (%saturation of Hgb)
NNN (N-nitrosonornicotine)—Tobacco Specific Nitrosamine	0.7–23 µg/m^3^	Urine NNN levels Urine NNN-Gluc levels

Table adapted from: (4) *Second Hand Tobacco Smoke.* IARC Monographs. International Agency for Research on Cancer. WHO. 2004; (5) Chang et al. *Biomarkers of Tobacco Exposure: Summary of an FDA-Sponsored Public Workshop.* Cancer Epidemiol Biomarkers Prev. 2017 Mar; 26(3): 291–302.

**Table 2:** Acute Effects of ETS on Clinical Parameters in Healthy Subjects.

Study	Heart Rate	Blood Pressure	Skin Temperature (°C)
Luguette et al., 1970 (*N* = 40) [45]	Before ETS: 89 After ETS: 97	Before ETS: 116/67 After ETS: 120/72	Not studied
Harke and Bleichert, 1972 (*N* = 10) [46]	Before ETS: 72 ± 8 After ETS: 74 ± 12	Before ETS: 123/84 After ETS: 121/84	Before ETS: 0.0 degrees After ETS: 0.0273 degrees
Rummel et al., 1975 (*N* = 56) [47]	Before ETS: 72 ± 10 After ETS: 71 ± 11	Before ETS: 117/71 After ETS: 117/71	Not studied
Hurshman et al., 1978 (*N* = 8) [48]	Before ETS: 73 After ETS: 79	Before ETS 107/67 After ETS: 114/68	Not studied
Pimm et al., 1978 (*N* = 20) [49]	Before ETS: 84 (F), 77 (M) After ETS: 80 (F), 80 (M)	Not studied	Not studied
